# Human Microglia Synthesize Neurosteroids to Cope with Rotenone-Induced Oxidative Stress

**DOI:** 10.3390/antiox12040963

**Published:** 2023-04-19

**Authors:** Chiara Lucchi, Alessandro Codeluppi, Monica Filaferro, Giovanni Vitale, Cecilia Rustichelli, Rossella Avallone, Jessica Mandrioli, Giuseppe Biagini

**Affiliations:** 1 Department of Biomedical, Metabolic and Neural Sciences, University of Modena and Reggio Emilia, 41125 Modena, Italy; lucchi.chiara86@gmail.com (C.L.); monica.filaferro@unimore.it (M.F.); jessica.mandrioli@unimore.it (J.M.); 2 Department of Life Sciences, University of Modena and Reggio Emilia, 41125 Modena, Italy; alle.codeluppi@gmail.com (A.C.); giovanni.vitale@unimore.it (G.V.); cecilia.rustichelli@unimore.it (C.R.); rossella.avallone@unimore.it (R.A.); 3 Department of Neurosciences, Ospedale Civile di Baggiovara, Azienda Ospedaliero-Universitaria di Modena, 41126 Modena, Italy

**Keywords:** allopregnanolone, microglia, neuroinflammation, neurosteroids, reactive oxygen species, rotenone

## Abstract

We obtained evidence that mouse BV2 microglia synthesize neurosteroids dynamically to modify neurosteroid levels in response to oxidative damage caused by rotenone. Here, we evaluated whether neurosteroids could be produced and altered in response to rotenone by the human microglial clone 3 (HMC3) cell line. To this aim, HMC3 cultures were exposed to rotenone (100 nM) and neurosteroids were measured in the culture medium by liquid chromatography with tandem mass spectrometry. Microglia reactivity was evaluated by measuring interleukin 6 (IL-6) levels, whereas cell viability was monitored by the 3-(4,5-dimethylthiazol-2-yl)-2,5-diphenyltetrazolium bromide assay. After 24 h (h), rotenone increased IL-6 and reactive oxygen species levels by approximately +37% over the baseline, without affecting cell viability; however, microglia viability was significantly reduced at 48 h (*p* < 0.01). These changes were accompanied by the downregulation of several neurosteroids, including pregnenolone, pregnenolone sulfate, 5α-dihydroprogesterone, and pregnanolone, except for allopregnanolone, which instead was remarkably increased (*p* < 0.05). Interestingly, treatment with exogenous allopregnanolone (1 nM) efficiently prevented the reduction in HMC3 cell viability. In conclusion, this is the first evidence that human microglia can produce allopregnanolone and that this neurosteroid is increasingly released in response to oxidative stress, to tentatively support the microglia’s survival.

## 1. Introduction

Neurosteroids are a family of molecules produced in the brain by metabolizing pregnenolone or converting peripherally synthesized steroids [1]. Allopregnanolone is considered the major representative steroid of the family and displays neuromodulatory properties mediated by membrane receptors, such as the γ-aminobutyric acid type A receptor (GABA_A_) [2]. Apart from generating chloride inhibitory currents in neurons, GABA_A_ has been proposed to mediate some anti-inflammatory properties of allopregnanolone [3]. However, the GABA_A_ mechanism appeared to be just one of those possibly involved in modulating the anti-inflammatory properties of neurosteroids [4]. Additionally, allopregnanolone has been shown to possess neuroprotective properties [5], and a reduction in allopregnanolone availability has been implied in various neurological disorders, including multiple sclerosis [6], Parkinson’s disease [7], Alzheimer’s disease [8], and epilepsy [9].

Although the presence of neurosteroids in the human brain has been well established in early studies using radioimmunoassays [10,11,12], the sources of human neurosteroids are still poorly characterized, and the current knowledge is mainly based on information from in vitro studies with human cell lines [13,14] and postmortem analyses of supposedly healthy donors or patients with different neurological disorders [15,16,17]. Based on these experiments, it has been suggested that neurosteroids are produced in the human brain thanks to oligodendrocytes that synthesize and release pregnenolone to fuel the production of other cognate molecules by neurons and astrocytes [18,19]. In particular, evidence supporting the synthesis of allopregnanolone in the human brain has been obtained by experiments using surgical specimens from patients with epilepsy [20]. However, it is undetermined which human brain cell type may produce allopregnanolone, although in rats, neurons have been indicated as the probable source of this neurosteroid [21].

Mouse microglia were found to express some steroidogenic enzymes capable of metabolizing dehydroepiandrosterone and androstenediol [22]. Subsequently, evidence was obtained to suggest that human microglia can produce pregnenolone, but it remained undetermined if other neurosteroids could be metabolized by these cells [14,23]. This is a relevant issue because microglia are one of the possible targets involved in the beneficial properties of allopregnanolone, which could be released by microglia to also exert autocrine effects. Recently, allopregnanolone properties have been characterized in cultured BV2 microglial cells and also in primary microglia cultures, disclosing an array of modulatory effects on phagocytosis and morphology, which markedly changed in the experimental conditions able to reproduce the interruption of the blood–brain barrier functions, as it occurs in several neurological disorders characterized by neuroinflammation [24]. Indeed, microglia are a major player not only in protecting the brain from aggression by pathogens [25], but also in mediating neuroinflammation [26], especially when the inflammatory process becomes chronic [27].

In the presence of a lesion, microglia become reactive, which means that microglial cells change their morphology to become ameboid with shorter and thicker pseudopodia and increase their expression of an array of proteins [28], of which the most popular to characterize their reactivity is the ionized calcium-binding adapter molecule 1 (Iba1) [29]. Indeed, the most important products synthesized and released to promote neuroinflammation by reactive microglia include interleukins (ILs), such as IL-1β and IL-6, tumor necrosis factor-α, interferon-γ, chemokine (C-C motif) ligand 2, chemokine (C-X3-C motif) ligand 1, and the C-X-C motif chemokine ligand 10; also, glutamate and nitric oxide are released by microglia during inflammation to promote tissue damage [30].

Rotenone is an inhibitor of the mitochondrial complex I, which produces oxidative damage [31]. This property has been exploited to reproduce features of Parkinson’s disease both in vitro and in vivo [32]. We recently described the cytotoxic effects of rotenone at 100 nM on cultured mouse BV2 microglial cells, which were related to the induction of reactive oxygen species (ROS) able to reduce microglia survival by 50% [33]. In such conditions, we described a profound derangement of neurosteroid metabolism, which was characterized by a remarkable reduction in pregnenolone sulfate and allopregnanolone levels and, at variance, increased levels of 5α-dihydroprogesterone (5α-DHP) and pregnanolone. These findings represented the first evidence of the capability to synthesize neurosteroids by murine microglia, but it is unknown whether human microglia could operate similarly, as questioned by other investigators who found that human microglia cell lines were able to produce pregnenolone [23].

Thus, in the present experiments, we aimed at evaluating if the human microglia could also be able to synthesize neurosteroids and if this property may be modified by challenging microglial cells with rotenone at the same dose used in our previous work to induce ROS production and cell death [33]. To disclose the activating effects of rotenone on the human microglial clone 3 (HMC3) cell line, we evaluated the changes in IL-6 levels and ROS in the culture medium. We also assessed HMC3 viability after 24 h (h) and 48 h of exposure to rotenone and the modifying effects of allopregnanolone added to cell cultures. These experiments revealed that human microglia can produce neurosteroids and that this property can be modified by rotenone-induced oxidative stress.

## 2. Materials and Methods

### 2.1. Chemicals and Reagents

Eagle’s minimum essential medium (EMEM) was purchased from ATCC (Manassas, VA, USA). Penicillin, streptomycin, fetal bovine serum (HyClone Laboratories, Logan, UT, USA), 3-(4,5-dimethylthiazol-2-yl)-2,5-diphenyltetrazolium bromide (MTT), rotenone, and the human IL-6 enzyme-linked immunosorbent assay (ELISA) kit were purchased from Merck Life Science (RAB0306, Milan, Italy); 2,7 dichlorodihydrofluorescein diacetate (H2DCFDA) was from Invitrogen (Thermo Fisher Scientific, Waltham, MA, USA); Hank’s Balanced Salt Solution (HBSS) was obtained from Gibco (Thermo Fisher Scientific, Waltham, MA, USA). All other chemical reagents were HPLC grade. The certified standard for the investigated neurosteroids and the isotope-labeled internal standards (allopregnanolone-2,2,3,4,4-d5 and pregnenolone-20,21-13C2-16,16-d2 sulfate sodium salt) were supplied as pure substances or solutions (100 µg/mL) from Merck Life Science. AmplifexTM-Keto Reagent Kit and Discovery DSC-18 SPE cartridges (100 mg; 1 mL) were also purchased from Merck Life Science. Acetonitrile (ACN), methanol, formic acid (FA), and ammonium formate (AmmF) were of liquid chromatography–mass spectrometry (LC-MS) purity grade (Merck Life Science); ultra-pure water was obtained by a Milli-Q Plus185 system (Millipore, Milford, MA, USA).

### 2.2. Cell Culture

HMC3 microglia (ATCC, Manassas, VA, USA)were grown in EMEM supplemented with 100 U/mL penicillin, 10 μg/mL streptomycin, and 10% fetal bovine serum (FBS) and kept in a humidified incubator at 37 °C with 95% O_2_/5% CO_2_. For all experiments, cells were grown to 80–90% confluency and then subjected to no more than 20 cell passages.

### 2.3. IL-6 Quantification

To evaluate the production and release of IL-6, we exposed the HMC3 cells to rotenone (100 nM) for 24 h and 48 h. For quantification, we used the IL-6 ELISA kit. Briefly, 100 μL of each standard and samples (culture media) were added to wells of a 96-well plate. The plate was covered and incubated for 2.5 h at room temperature. After discarding the standard and samples, the plate was cleaned four times with a washing solution. After that, the detection antibody (100 μL) was added to each well, and the plate was incubated at room temperature for 1 h with gentle shaking. Following four washes with washing solution, 100 μL of horseradish peroxidase–streptavidin was added to each well. The plate was subsequently incubated for 45 minutes (min) at room temperature and washed four times with the washing solution. After that, 100 μL of the ELISA colorimetric 3,3,5,5–tetramethylbenzidine reagent was added to each well for 30 min at room temperature and covered. A total of 50 μL of stop solution was added, and the plate was read at 450 nm immediately on the microplate reader (Multiskan FC Microplate, Thermo Fisher Scientific, Waltham, MA, USA). All the values obtained with the ELISA assay were normalized with the total protein content of each sample (cell lysate according to the Bradford method).

### 2.4. Determination of ROS

The ROS generation was determined using the fluorogenic probes H2DCFDA according to the manufacturer’s instructions. Briefly, HMC3 cells were seeded in a 96-well plate (at a density of 10,000 cells/well) and maintained in complete culture media for 24 h. Afterward, cells were washed once with HBSS and incubated for 45 min at 37 °C with 10 µM H2DCFDA dye. After incubation, the dye was removed and cells were treated for 24 h with rotenone 100 nM, control medium, or H_2_O_2_ used as a positive control. Cell staining was performed in HBSS. The emitted fluorescence intensity was measured using a Fluoroskan FL Microplate Fluorometer (Thermo Fisher Scientific, Waltham, MA, USA) with wavelengths of 485 nm (excitation) and 520 nm (emission).

### 2.5. Sample Processing for Neurosteroid Quantification

A total of 100,000 HMC3 cells/well were seeded in 24 well plates in serum-free Roswell Park Memorial Institute (RPMI) 1640 medium without phenol red for the neurosteroid quantification. Then, cells were treated with rotenone (100 nM) for at least 24 h. As previously published [33], the medium was aspirated and centrifuged at the end of treatment to remove cells in suspension, and the supernatant was used to process the neurosteroid analysis. Aliquots (700 µL) of cell medium were spiked with 50 µL of the internal standard solution, vortexed (90 s, s), and purified using the C-18 SPE procedure. The obtained eluates were dried using an Eppendorf Concentrator Plus AG5305 (Eppendorf AG, Hamburg, Germany) and derivatized with Amplifex Keto Reagent (50 µL) for 1 h at room temperature and in the dark. Finally, the obtained samples were resuspended with 50 µL methanol/water (70/30), centrifuged, and analyzed using LC-MS/MS.

### 2.6. Working Solutions and Calibrators

To obtain the working solutions at 10 concentration levels, a stock solution containing all of the examined neurosteroids was serially diluted with methanol. Moreover, a stock solution of the isotope-labeled internal standards (ISs) was prepared in methanol at a concentration of 1000 fg/mL for both ISs. All solutions were kept at −20 °C until use. To obtain calibration samples (*n* = 10) in the range of 5.0 ± 1250 fg/700 µL for pregnenolone and 5α-DHP, 1.0 ± 250 fg/700 µL for pregnanolone, and 0.2 ± 50 fg/700 µL for pregnenolone sulfate, progesterone, and allopregnanolone; aliquots (700 µL) of blank cell medium were spiked with 50 µL of the ISs solution and 50 µL of the working solutions. The calibrators were prepared as previously reported [33] and evaluated in triplicate on three separate days.

### 2.7. LC-MS/MS Analysis

LC analyses were performed as described previously [33]. Briefly, we used a Kinetex XB-C18 column (100 mm length × 2.1 mm inner diameter; 2.6 µm particle size) equipped with a UHPLC C18 SecurityGuard cartridge (2.1 mm) (Phenomenex, Torrance, CA, USA). Mass spectrometric detection was performed using an Agilent QQQ-MS/MS (6410B) triple quadrupole operating in electrospray positive ionization mode. In the case of progesterone and 5α-DHP, the presence of two keto groups led to cis/trans derivatives, which eluted as two separable LC peaks; therefore, to quantitate progesterone and 5α-DHP in medium samples, the summation of both peaks was used.

### 2.8. Viability of HMC3 Microglial Cells Treated with Rotenone

To evaluate the viability of HMC3 cells exposed to rotenone (100 nM), the 3-(4,5-dimethylthiazol-2-yl)-2,5-diphenyltetrazolium bromide (MTT) test was performed. The rotenone 100 nM concentration was selected because it was shown to be below IC_50_ in our previous experiment, with no progression in cell death over an observation period of 72 h [33]. A total of 10,000 HMC3 cells were seeded in 96 well plates. After 24 h, cells were exposed to rotenone or the combination of rotenone (100 nM) and allopregnanolone (1 nM) and incubated for 24 h and 48 h. Subsequently, the MTT solution was added to each well (10 µL) and incubated for 2 h. Finally, a 10% sodium dodecyl sulfate solution was added to dissolve formazan, and absorbance was measured at 570 nm, using 620 nm as a wavelength reference.

### 2.9. Statistical Analysis

Data were analyzed using SigmaPlot 11 (Systat Software, San Jose, CA, USA). The evaluation of IL-6 was carried out using the two-way analysis of variance (ANOVA) and Holm–Šídák as post hoc tests. Data analysis of ROS assay was compared using one-way ANOVA and Bonferroni post hoc test. The neurosteroid levels were compared using the Mann–Whitney test. The data on viability at 24 h were analyzed using the Student’s *t*-test. The data on viability at 48 h were compared using one-way ANOVA and Holm–Šídák as post hoc tests. A *p*-value < 0.05 was considered to be statistically significant. Results are illustrated by mean values and standard error of the mean (SEM) or median values and interquartile range.

## 3. Results

### 3.1. Oxidative Stress Activates HMC3 Microglia to Release IL-6

**Figure 1:**
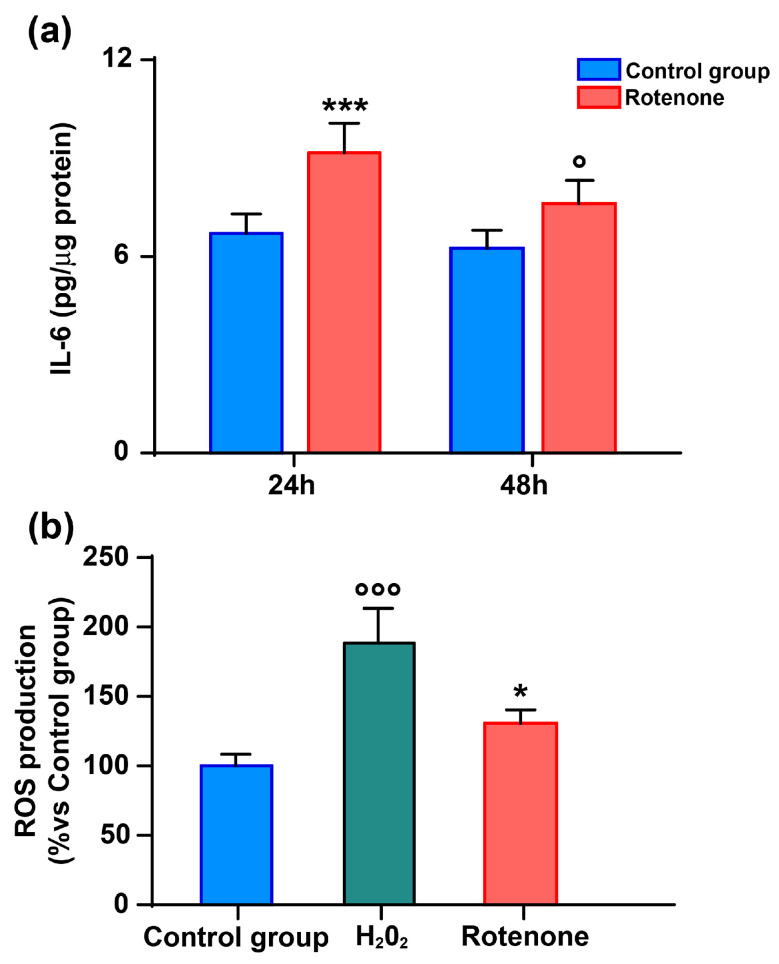
(**a**) Effect of oxidative stress on IL-6 production by HMC3 cells. After 24 h, the treatment with rotenone (100 nM) significantly increased the release of IL6 when compared to unstimulated cells. Furthermore, prolonged oxidative stress did not lead to additional IL-6 release but its levels remain significantly higher when compared to the control group. Each column represented the average ± SEM (*n* = 5) normalized by the total protein content measured per well. Data were analyzed by two-way ANOVA followed by Holm–Šídák post hoc test. ° *p* < 0.05 vs. control group 48 h; *** *p* < 0.001 vs. control group 24 h. In (**b**), bars illustrate the ROS production in HMC3 cells following rotenone treatment (100 nM). Each bar represents the mean ± SEM (*n* = 5) of the percentage of the fluorescence, normalized with the respective value of the control. Data were analyzed by one-way ANOVA followed by Bonferroni post hoc test. * *p* < 0.05 vs. control group; °°° *p* < 0.001 H_2_O_2_ vs. rotenone and control groups.

As previously shown by others [34] and illustrated in Figure 1, we confirmed that HMC3 cells can produce and release IL-6 in resting conditions. As expected, after 24 h of stimulation, rotenone activated the HMC3 microglia inasmuch as they produced a significant increase in the release of IL-6 (+37%) compared to the basal condition (*p* < 0.001, two-way ANOVA and Holm–Šídák post hoc test). On the other hand, prolonged exposure to rotenone did not lead to an additional release of IL-6, whose levels remained significantly higher when compared to the control group (*p* < 0.05, two-way ANOVA and Holm–Šídák post hoc test) (Figure 1a).

As already demonstrated for BV2 cells, rotenone led to a significant increase in ROS production in HMC3 after 24 h (*p* < 0.05 rotenone vs. controls; one-way ANOVA followed by Bonferroni post hoc test) using, this time, a more ubiquitous fluorogenic probe. However, this increase was significantly lower when compared to the changes in ROS levels caused by the exposure to strong oxidants, such as H_2_O_2_ (500 µM) (*p* < 0.001, H_2_O_2_ vs. control or rotenone levels, one-way ANOVA followed by Bonferroni correction) (Figure 1b).

### 3.2. Effect of Rotenone on Neurosteroid Levels in Culture Medium of HMC3 Cells

To evaluate if human microglia could produce neurosteroids, we measured pregnenolone, pregnenolone sulfate, progesterone, 5α-DHP, pregnanolone, and allopregnanolone by LC-MS/MS in the culture medium of HMC3 cells, both at rest and after 24 h of exposure to rotenone (100 nM).

**Figure 2:**
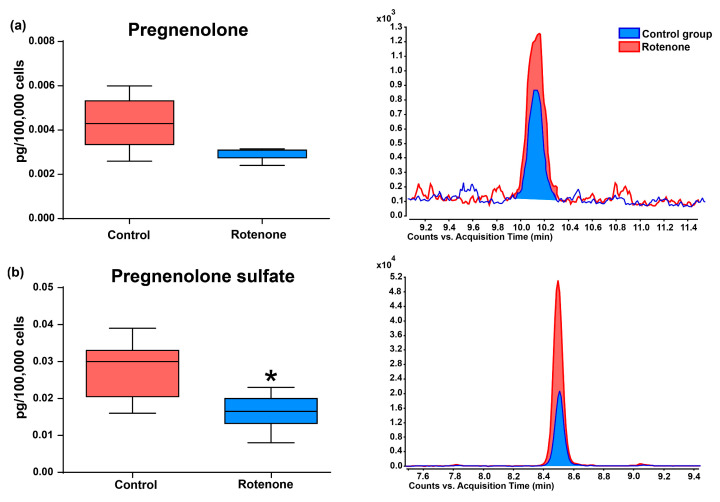
Pregnenolone (**a**) and pregnenolone sulfate (**b**) concentrations in HMC3 cells’ medium determined by LC-MS/MS in microglia in resting condition (Control) or after activation by exposure to rotenone for 24 h. In (**a**), pregnenolone levels, illustrated in the box plot, after stimulation with rotenone were lower but not significantly different when compared to the control group. Peak areas corresponding to the respective median values are illustrated on the right. In (**b**), pregnenolone sulfate levels, illustrated in the box plot, were significantly lower (* *p* < 0.05, Mann–Whitney rank sum test) after stimulation with rotenone. Peak areas corresponding to the respective median values are illustrated on the right. The neurosteroid concentrations are expressed in pg/100,000 cells.

**Figure 3:**
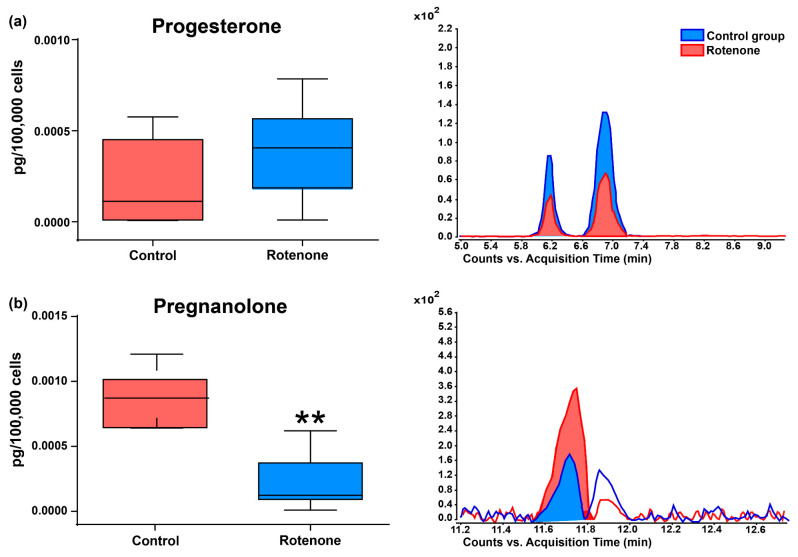
Progesterone (**a**) and pregnanolone (**b**) concentrations in HMC3 cells’ medium determined by LC-MS/MS in microglia in resting condition (Control) or after activation by exposure to rotenone for 24 h. In (**a**), progesterone levels, illustrated in the box plot, after stimulation with rotenone increased but not significantly when compared to the control group. Peak areas corresponding to the respective median values are illustrated on the right. In (**b**), pregnanolone levels, illustrated in the box plot, were significantly lower (** *p* < 0.01, Mann–Whitney rank sum test) after stimulation with rotenone. Peak areas corresponding to the respective median values are illustrated on the right. The neurosteroid concentrations are expressed in pg/100,000 cells.

**Figure 4:**
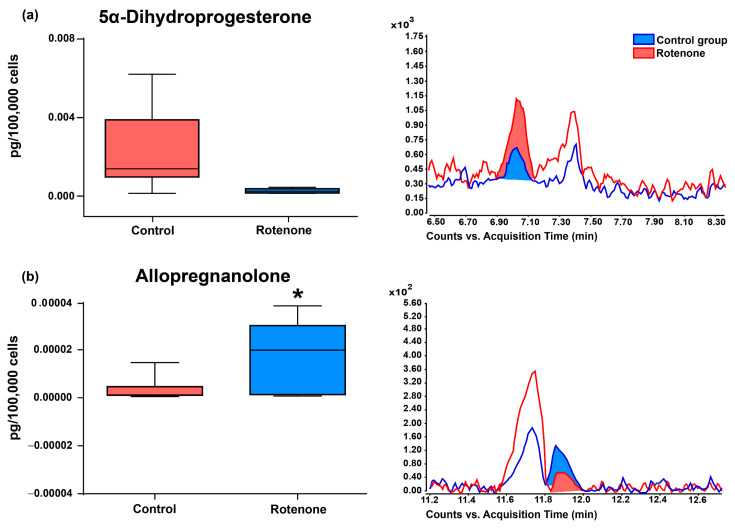
5α-Dihydroprogesterone (5α-DHP) (**a**) and allopregnanolone (**b**) concentrations in HMC3 cells’ medium determined by LC-MS/MS in microglia in resting condition (Control) or after activation by exposure to rotenone for 24 h. In (**a**), 5α-DHP levels, illustrated in the box plot, after stimulation with rotenone were lower but not significantly different when compared to the control group. Peak areas corresponding to the respective median values are illustrated on the right. In (**b**), allopregnanolone levels, illustrated in the box plot, were significantly reduced (* *p* < 0.05, Mann–Whitney rank sum test) after stimulation with rotenone. Peak areas corresponding to the respective median values are illustrated on the right. The neurosteroid concentrations are expressed in pg/100,000 cells.

First, we established that all the evaluated neurosteroids were detectable, although in low amounts, in basal conditions (Figure 2, Figure 3 and Figure 4). In particular, pregnenolone sulfate was the most synthesized neurosteroid (about 0.03 pg/100,000 cells), followed by pregnenolone (at 0.004 pg/100,000 cells). Lower amounts were also found for pregnanolone (approximately 0.008 pg/100,000 cells), 5α-DHP (0.002), progesterone (0.0001 pg/100,000 cells), and, especially, allopregnanolone, which was barely detectable (0.000002 pg/100,000 cells).

This scenario was completely modified by the treatment of HMC3 cells with rotenone. Rotenone induced a remarkable reduction in pregnenolone sulfate levels with respect to the basal condition (*p* < 0.01, Mann–Whitney rank sum test; Figure 2b). In addition, pregnenolone levels were slightly decreased in comparison to the control group (*p* = 0.052; Figure 2a).

Progesterone levels increased but not significantly (*p* = 0.310; Figure 3a). Pregnanolone levels were highly reduced by rotenone treatment (−85%), especially when rotenone-exposed cells were compared to the unstimulated microglia (*p* < 0.01; Figure 3b).

Levels of 5α-DHP were only slightly reduced in the medium of rotenone-treated HMC3 (*p* = 0.052; Figure 4a). Notably, the main progesterone metabolite, allopregnanolone, presented a remarkable increase in response to rotenone treatment (*p* < 0.05 vs. unstimulated cells; Figure 4b).

### 3.3. Exogenous Allopregnanolone Preserved HMC3 Cell Viability from Rotenone-Induced Damage

**Figure 5:**
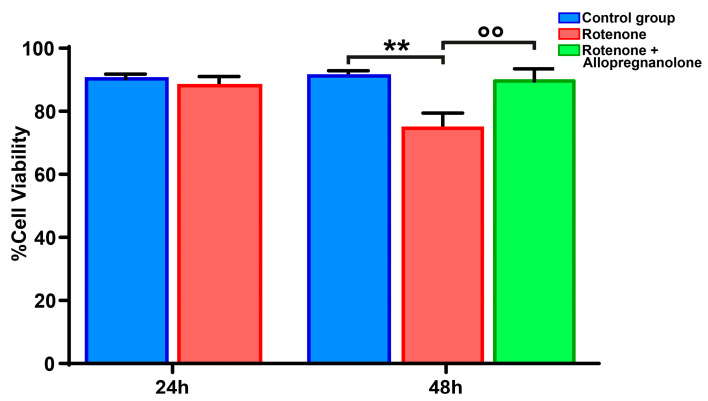
Viability of HMC3 cells exposed to rotenone (100 nM) at different time intervals (24 h and 48 h) and rescued by allopregnanolone (1 nM). The data represent the average ± SEM of two independent experiments. ** *p* < 0.01 rotenone vs. control group; °° *p* < 0.01 rotenone + allopregnanolone vs. rotenone, according to one-way analysis of variance (ANOVA) followed by Holm–Šídák post hoc test for multiple comparisons.

We evaluated whether rotenone could influence the survival of HMC3 microglia using the MTT test, as we previously found a cytotoxic effect on murine microglia [33]. At variance with the previous results, exposure to rotenone (100 nM) did not affect HMC3 cell viability (control condition: 90 ± 1.5% vs. rotenone treatment: 88 ± 2.8%) after 24 h (Figure 5), but significantly decreased the survival of human microglia after 48 h (*p* < 0.01, control condition: 97 ± 4.5% vs. rotenone treatment: 74 ± 4.7%), even if at a lower extent in respect to BV2 cells [33].

Since we interpreted the difference observed at 24 h in the response to rotenone of HMC3 vs. BV2 microglia as possibly related to the altered allopregnanolone metabolism, which led to reduced allopregnanolone levels in BV2 [33], we hypothesized that it could be possible to limit the HMC3 cell death found at the 48 h time interval by further increasing allopregnanolone in the culture medium. To this aim, we decided to combine rotenone treatment with allopregnanolone, exogenously added to HMC3 cultures at 1 nM; thus, we observed that HMC3 cell viability was maintained at values similar to those of the control condition (*p* < 0.01, Holm–Šídák post hoc test; rotenone treatment: 74 ± 4.7% vs. rotenone + allopregnanolone treatment: 93.8 ± 5.4%).

## 4. Discussion

We previously found that murine microglia were able to produce and release a variety of neurosteroids other than pregnenolone in BV2 cell cultures [33] and that this property could be markedly modified by oxidative stress able to affect cell survival. Indeed, our previous work elicited some criticism, which we already mentioned in the introduction. Precisely, Germelli and collaborators [23] highlighted that the ability of BV2 cells to produce neurosteroids was a novel finding requiring confirmation in the human microglia so as to possibly design new therapeutic approaches to neurological disorders, especially when characterized by neuroinflammation. Thus, in the present work, we explored the possibility that: (i) human microglia could be able to synthesize and release neurosteroids; (ii) synthesis of these steroidal molecules could be modified in response to oxidants such as rotenone. We also evaluated the possibility that the major neurosteroid, allopregnanolone, could produce a protective effect on human microglia exposed to the toxic activity of rotenone. The main findings of our study show that also the human microglia is able to produce and release neurosteroids, as previously found for the mouse microglia [33], and that this ability could be altered by neurotoxicants such as rotenone. Furthermore, as an additional novel finding, we showed that a high dose of exogenous allopregnanolone could result in rescue effects for the human microglia endangered by metabolic challenges, as in the case of exposure to rotenone, thus suggesting a possible therapeutic use of this neurosteroid to protect the microglia.

Although HMC3 microglia were able to produce neurosteroids in basal conditions, the metabolic profile of the evaluated molecules was different from that found in mouse BV2 cells [33]. Indeed, in our previous study, we found that neurosteroids were present at much higher levels in the culture medium of BV2 cells. For instance, pregnenolone was at a concentration 500-fold higher than that found in the HMC3 culture medium. Consequently, pregnenolone sulfate was also approximately 67-fold higher in the BV2 culture medium. Similar or even more remarkable differences could be mentioned for the other neurosteroids, including progesterone, 5α-DHP, and allopregnanolone, with the only exception of pregnanolone, which was produced at very low levels also by resting BV2 cells.

This scenario was notably changed by rotenone, which induced in HMC3 culture medium changes that were partly different from those observed for BV2 cells. Despite the fact that a 24 h exposure to rotenone did not significantly modify the levels of pregnenolone, pregnenolone sulfate, progesterone, 5α-DHP, pregnanolone, and allopregnanolone in the mouse BV2 cell cultures, human HMC3 microglia responded to rotenone by reducing the levels of pregnenolone sulfate and pregnanolone and by increasing the levels of allopregnanolone. Thus, human microglia appeared to react more rapidly to oxidative damage caused by rotenone than mouse microglia, which instead modified the release of neurosteroids in the culture medium only 48 h after the rotenone treatment [33]. Interestingly, the late changes in neurosteroid release of BV2 cells were also qualitatively different from those evidenced in HMC3 cells because rotenone produced an increase in 5α-DHP and pregnanolone levels and reduced pregnenolone levels in the culture medium of BV2 cells with no changes in allopregnanolone levels. Thus, the ability to modify allopregnanolone metabolism in response to the oxidant rotenone appears to be specific for the human microglia, at least in the case of HMC3 cells. Alternatively, it could be proposed that the differences in allopregnanolone levels in HMC3 and BV2 cell cultures were dependent on the different extent of rotenone toxicity, which was more pronounced for BV2 microglia, but this interpretation is at odds with the changes observed for the other neurosteroids, 5α-DHP and pregnanolone, which increased in the BV2 culture medium.

Allopregnanolone is a protective molecule [5], but the changes we observed in response to rotenone were ineffective in promoting the survival of HMC3 cells for more than 24 h. However, by adding allopregnanolone exogenously, we were able to block the detrimental effects of rotenone on the survival of HMC3 cells, as observed at 48 h. The extremely increased concentration of allopregnanolone required to obtain this beneficial result may explain why the changes in allopregnanolone synthesis and release by HMC3 microglia were not sufficient to afford protection at the 48 h time interval. Anyway, the effective allopregnanolone concentration was close to the range of levels measured in the cerebrospinal fluid of healthy humans (females: 0.09 nM; males: 0.2 nM) [35], suggesting that the contribution of microglia to neurosteroid synthesis may be limited because other sources can provide adequate levels of allopregnanolone to protect microglial cells. Moreover, allopregnanolone concentration could be increased in the brain tissue by drugs that modify the steroid metabolism in the adrenal gland, such as trilostane [36], to pave the way for the growing field of microglia pharmacology [37].

We did not investigate any possible mechanisms activated by allopregnanolone to afford protection from the oxidative effects of rotenone. Indeed, this is a limitation of our study, and further experiments will be required to address this issue. Interestingly, an in vivo study using mice treated with pilocarpine to induce a status epilepticus evidenced a reduction in ROS cerebral tissue levels and oxidative damage by treatment with allopregnanolone. This effect was associated with an increased expression of superoxide dismutase 2 in the hippocampus of allopregnanolone-treated mice [38]. It is also interesting to note that the oxidative effect of silver nanoparticles disrupting the antioxidant defense in the hippocampus of Wistar rats was associated with a reduction in progesterone, 17α-hydroxyprogesterone, and testosterone hippocampal levels, whereas allopregnanolone, dehydroepiandrosterone, dehydroepiandrosterone sulfate, androstenedione, dihydrotestosterone, and 17β-estradiol hippocampal levels were increased or unmodified, depending on the silver formulation used [39]. It is also worth noting that both progesterone and allopregnanolone exerted beneficial effects in the Wobbler mouse model of amyotrophic lateral sclerosis by preserving mitochondrial respiratory complex I activity, reducing the mitochondrial expression and activity of nitric oxide synthase, and inducing the Mn-dependent superoxide dismutase [40].

## 5. Conclusions

Our work provides the first evidence that human microglia produce and release neurosteroids and that this metabolic activity could be modulated in response to a damaging event. We also found that the anti-inflammatory neurosteroid allopregnanolone could provide microglia protection at physiological levels [41], which might be further increased by pharmacological approaches. These results constitute a pathophysiological background to encourage the investigators to consider neurosteroids and, especially, allopregnanolone as a possible useful therapeutic tool to cope with brain diseases in which oxidative stress and neuroinflammation play a pathological role, as in the case of multiple sclerosis, amyotrophic lateral sclerosis, Parkinson’s disease, Alzheimer’s disease, and epilepsy.

## Data Availability

The data presented in this study are available on request from the corresponding author.
